# Relationships between Viral Load and the Clinical Course of COVID-19

**DOI:** 10.3390/v13020304

**Published:** 2021-02-15

**Authors:** Hiroyuki Tsukagoshi, Daisuke Shinoda, Mariko Saito, Kaori Okayama, Mitsuru Sada, Hirokazu Kimura, Nobuhiro Saruki

**Affiliations:** 1 Gunma Prefectural Institute of Public Health and Environmental Sciences, Maebashi-shi, Gunma 371-0052, Japan; shinoda-daisuke@pref.gunma.lg.jp (D.S.); saito-mariko@pref.gunma.lg.jp (M.S.); saruki-n@pref.gunma.lg.jp (N.S.); 2 Department of Health Science, Gunma Paz University Graduate School of Health Sciences, Takasaki-shi, Gunma 370-0006, Japan; okayaman0811@std.kyorin-u.ac.jp (K.O.); rainbow_orchestra716@yahoo.co.jp (M.S.); kimhiro@nih.go.jp (H.K.); 3 Department of Respiratory Medicine, Kyorin University School of Medicine, Mitaka-shi, Tokyo 181-8611, Japan

**Keywords:** coronavirus disease-2019 (COVID-19), viral load, clinical course

## Abstract

To predict the clinical outcome of coronavirus disease-2019 (COVID-19), we examined relationships among epidemiological data, viral load, and disease severity. We examined viral loads of severe acute respiratory syndrome coronavirus type 2 (SARS-CoV-2) in fatal (15 cases), symptomatic/survived (133 cases), and asymptomatic cases (138 cases) using reverse transcription quantitative real-time PCR (RT-qPCR). We examined 5768 nasopharyngeal swabs (NPS) and attempted to detect the SARS-CoV-2 genome using RT-qPCR. Among them, the viral genome was detected using the method for the 370 NPS samples with a positive rate of 6.4%. A comparison of each age showed that the fatal case was higher than the survived case and asymptomatic patients. Survived cases were older than asymptomatic patients. Notably, the viral load in the fatal cases was significantly higher than in symptomatic or asymptomatic cases (*p* < 0.05). These results suggested that a high viral load of the SARS-CoV-2 in elderly patients at an early stage of the disease results in a poor outcome. We should, therefore, intervene early to prevent a severe stage of the disease in such cases.

## 1. Introduction

A new coronavirus disease-2019 (COVID-19) caused by severe acute respiratory syndrome coronavirus type 2 (SARS-CoV-2) occurred as a pandemic resulting in serious disease burden in almost all countries [1,2]. To detect and diagnose the virus, reverse transcription quantitative real-time polymerase chain reaction (RT-qPCR) was applied in many countries [3]. Also, this method can estimate the viral load in samples from patients with this viral infection [3]. For example, using RT-qPCR, the viral load in respiratory specimens, such as throat swab and nasopharyngeal swabs (NPS) of patients with seasonal influenza have been reported [4]. Various factors related to mortality in COVID-19 have been investigated. This indicated an association with mortality, such as age, underlying diseases, IL-6, and D-dimer [5,6]. However, the relationships between epidemiological data, viral load, and clinical course of COVID-19 may not be exactly known. Therefore, we analyzed relationships among them in this study.

## 2. Materials and Methods

### 2.1. Demographic Data, Samples, and Reverse Transcription Quantitative Real-Time PCR (RT-qPCR)

Table 1 shows the demographic data. Here, the sample collected was classified into three groups, including fatal cases (15 cases), symptomatic/survival cases (symptomatic cases, 133 cases), and asymptomatic cases (138 cases). We collected samples from fatal and symptomatic cases within five days of the onset of symptoms such as fever, cough, sore throat, upper respiratory inflammation or pneumonia, or both. Most of the asymptomatic cases were closely contacted the patients with COVID-19 [7].

At the time of specimen collection, NPS specimens were collected from cases showing COVID-19 suspected symptoms, such as fever, sore throat, and cough or symptomatic individuals in Gunma Prefecture, Japan. From 15 February to 31 October 2020, 5768 NPS samples were collected. NPS specimens were centrifuged at 3000 rpm for 15 min. Viral RNA was extracted using an available kit (Qiagen, Hilden, Germany) [8].

One-step reverse transcription quantitative real-time PCR (RT-qPCR) was performed as previously described [8]. Primer and probe sequences were as follows: 

NIID_2019-nCOV_N_F2: 5′-AAATTTTGGGGACCAGGAAC-3′; 

NIID_2019-nCOV_N_R2: 5′-TGGCAGCTGTGTAGGTCAAC-3′; 

NIID_2019-nCOV_N_P2: 5′-FAM-ATGTCGCGCATTGGCATGGA-BHQ-3′. One-step RT-qPCR amplification was performed using a 7500 fast real-time PCR system (Applied Biosystems, Waltham, MA, USA) under the following conditions: reverse transcription 50 °C for 30 min and denaturation of UNG at 95 °C for 15 min to activate DNA polymerase, then 45 cycles of amplification with denaturalization at 95 °C for 15 s, and annealing and extension at 60 °C for 1 min. Amplification data were collected and analyzed using the sequence detector software v.2.0.6 (Applied Biosystems).

### 2.2. Ethical Statement

To diagnose COVID-19, all samples were primarily collected under compliance with the Act on the Prevention of Infectious Diseases and Medical Care for Patients with Infectious Diseases of Japan. All subjects were given orally with informed consent, which was obtained from the subjects or their legally acceptable representatives for sample donation. The patients’ data were anonymized. To perform this extraneous study (this study) and due to the lack of written informed consent, the present study protocols were deliberated by the Ethics Committee on Human Research of the Gunma Paz University (Gunma, Japan). Finally, this study was confirmed to have no infringement of the patient’s rights and was approved by the committee (approved no. PAZ20-22). Also, all methods were performed in accordance with the approved guidelines.

### 2.3. Statistical Analysis

Data were analyzed using SPSS software (SPSS for Windows, v.10.0). Comparison among groups was performed using the one-way analysis of variance and the Bonferroni’s multiple comparison tests. The chi-square test was used to evaluate whether there is an association between body temperature or pneumonia and patients’ prognosis. Statistical significance was set at *p* < 0.05.

## 3. Results

### 3.1. Positive Rate and Demographic Data in Each Group

**Figure 1:**
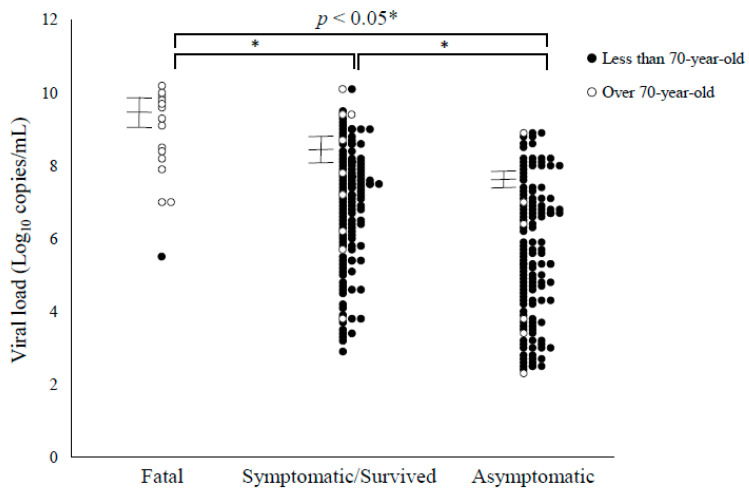
Viral load of SARS-CoV-2 in the present cases. Black circles, less than 70-year-old cases; White circles, over 70-year-old cases. Error bars represent 95% confidence intervals.

From 15 February to 31 October 2020, 5768 NPS samples were collected, and an attempt to detect the SARS-CoV N gene using RT-qPCR was performed. As a result, the viral genome was detected using the method for the 370 NPS samples with a positive rate of 6.4% (370/5768). Among them, virus positives within five days from the date of onset were as follows: 15 fatal cases (5.2%, 15/286), 133 symptomatic/survived cases (46.5%, 133/286), and 138 were asymptomatic (48.3%, 138/286) (Table 1). A comparison of each age showed that the fatal case was higher than the survived case and asymptomatic patients. Although survived cases were older than asymptomatic patients. Alternatively, there was no significant difference in gender. In this study, we did not trace the outcome of asymptomatic cases. Other demographic data were shown in Figure 1. Of them, pneumonia was significantly associated with mortality.

### 3.2. Viral Loads in the Present Cases

In the present cases, we compared SARS-CoV-2 viral loads in the NPS samples. Detailed data are shown in Figure 1. Comparing the number of viral copies at the time of sample collection, 3.57 × 10^9^ ± 4.70 × 10^9^ copies/mL (mean ± standard deviation (SD), 95% CIs (confidence intervals), 1.19 × 10^9^–5.95 × 10^9^) in the fatal cases, 3.92 × 10^8^ ± 1.60 × 10^9^ copies/mL (mean ± SD, 95% CIs, 1.20 × 10^8^–6.64 × 10^8^) in survived cases, and 4.92 × 10^7^ ± 1.48 × 10^7^ copies/mL (mean ± SD, 95% CIs 2.45 × 10^7^–7.38 × 10^7^) in asymptomatic cases. These results suggest that the viral loads of the fatal and symptomatic/survived cases were higher than asymptomatic cases (Figure 1). 

## 4. Discussion

Here, we investigated the relationships among epidemiological data, viral load, and disease severity in SARS-CoV-2 infections. Within five days of disease onset, our results suggest that the viral load in the fatal cases was significantly higher than in symptomatic or asymptomatic cases (*p* < 0.05). Also, the age of the fatal cases was significantly greater than the symptomatic and asymptomatic cases. In contrast, there was no significant difference in the viral load between patients aged over and under 70 years in each group. Although high fever (≥38°C) was not significantly associated with mortality, at the time of the virus test, patients in fatal cases showed pneumonia symptoms more than survived cases. Thus, if patients with the COVID-19 (aged over 70 years) showed a high viral load of more than 1.0 × 10^9^ copies/mL and showed pneumonia within five days of disease onset, these cases resulted in a poor outcome in the present cases. These results may be first the observation and may contribute as an early indicator of disease management.

Some reports regarding relationships between viral loads and disease severity have been reported [9]. For example, Liu et al. showed that the viral load of severe cases was higher than in mild cases [10]. Magleby et al. reported that the risk of incubation and death increased with higher viral loads [11]. Moreover, Fajnzylber et al. revealed that viral load was implicated in the severity and mortality of COVID-19 [12]. These data were partly compatible with our data. Alternatively, Lee et al. showed that there was no significant difference between viral load and clinical course in a cohort study [13]. Argyropoulos et al. demonstrated viral load of inpatients with the disease was lower than in outpatients [14]. Furthermore, Zou et al. showed that the viral load between symptomatic and asymptomatic cases was similar, although relatively small numbers of cases were examined [15]. These differences may be due to the differences in study design and timing of sample collection. The viral load may change the disease phase; this may be a reason for the differences [16]. Bitker et al. suggested that sustained abrogation of type-I interferon production may be associated with longer viral shedding and disease severity [17]. However, it is unclear if these mechanisms result in a relationship between viral load and disease severity. Hereafter, further studies regarding the optimum phase of the sample collection and disease criteria may be required.

Next, previous reports showed the differences between the viral load, age, and disease severity [18]. This study showed that the viral load of elderly patients (> 60 years) was higher than younger patients [18]. This study also showed that the viral load and age in the fatal cases were significantly higher than in survived and asymptomatic cases, while no differences existed between age and viral load in survived cases. A report suggested that increased body temperature did not correlate with prognosis [19]. Pneumonia is the most common severe manifestation of COVID-19 [20]. Here, body temperature was not related to prognosis, but whether the patient showed pneumonia symptoms was important for prognosis. Altogether, in the early stages of COVID-19, cases of patients aged over 70 years with high viral excretion, especially pneumonia symptoms, may have a poor prognosis. This may be the first observation.

This study had some limitations. First, we could not follow up whether the asymptomatic cases subsequently developed the disease or not. Reports suggested that most asymptomatic cases did not onset, although the onset rates were different in each study [21,22]. Moreover, the viral load may fluctuate before and after the onset of the disease [23,24]. Thus, viral loads in the present asymptomatic cases may change after disease onset. Alternatively, Zheng et al. reported that the viral load in patients with severe disease continued to be high three to four weeks after disease onset [9]. Also, relatively small numbers of fatal cases (15 cases) were examined in this study. Further studies with a larger number of fatal cases are required. 

## 5. Conclusions

In conclusion, our results suggested that a high viral load (over 10^9^ copies) of the SARS-CoV-2 in elderly patients (more than 70-year-old) at an early stage of the disease, especially pneumonia symptom results in a poor outcome. Therefore, we should intervene early to prevent a severe stage of the disease in such cases.

## Figures and Tables

**Table 1:** Demographic data of the present study.

	All	Fatal	Symptomatic/Survived	Asymptomatic
No. of patients	286	15	133	138
Age (Mean ± SD)	39.0 ± 35.0	84.7 ± 8.5 ^a^	40.0 ± 21.3 ^b, c^	28.9 ± 20.0 ^d^
Sex (Male/Female)	161/125	10/5	73/60	78/60
Fever [>38 °C, (%)]	59 (20.6)	5 (33.3)	54 (40.6)	NA
Pneumonia (%)	16 (5.6)	8 (53.3)	8 ^b^ (6.0)	NA

*p* values less than 0.05 were considered statistically significant. NA: not applicable. SD: standard deviation. ^a^ Fatal case vs. Asymptomatic case. ^b^ Survived case vs. Fatal case. ^c^ Survived case vs. Asymptomatic case. ^d^ Asymptomatic case vs. Fatal case.

## Data Availability

Not applicable.
